# Neuroendocrine–Immune Systems Response to Environmental Stressors in the Cephalopod *Octopus vulgaris*

**DOI:** 10.3389/fphys.2016.00434

**Published:** 2016-09-28

**Authors:** Anna Di Cosmo, Gianluca Polese

**Affiliations:** Department of Biology, University of Napoli Federico IINapoli, Italy

**Keywords:** *Octopus vulgaris*, immune system, neuroendocrine system, cephalopods, environmental stressor

## Abstract

Under a continuous changing environment, animals are challenged with stresses and stimuli which demanding adaptation at behavioral and physiological levels. The adaptation strategies are finely regulated by animal nervous, endocrine, and immune systems. Although it's been established by now the usage of integrative approach to the study the endocrine and nervous systems (neuroendocrine), yet our understanding of how they cooperate with the immune system remains far from complete. The possible role that immune system plays as a component of the network has only been recognized recently. *Octopus vulgaris* is an important member of cephalopods and is considered as a model species, with considerable information about the neuroendocrine and immune systems. In the current review, we anticipate to shed light on the complexity and cross talk among the three systems and how they cooperate in setting physiological response to stresses-stimuli in *O. vulgaris* as a target species and primary example.

## Introduction

In aquatic and terrestrial environments, animals are continuously challenged by stressful stimuli, and in response to which, react to show a coordinated series of physiological changes (Kassahn et al., 2009). The brain/endocrine and immune systems represent the major internal correlation systems within the organisms (Tada, 1997; Elenkov et al., 2000). Although acting independently of one another, these systems communicate in an integrated way to coordinate a set of appropriate physiological and behavioral responses. In contrast to the well-established integrative approach to the study of the endocrine and nervous systems our understanding of how these three systems cooperate remains incomplete, due to the fact that immune system, as component of this network, has only recently been recognized (Demas et al., 2010). Now it is known that a variety of extrinsic and intrinsic factors can affect the regulatory network composed by three systems and they are deeply interconnected by a bi-directional communication as shown by numerous experiments in vertebrates (Blalock, 1989). However, many literatures suggest that some of the same neuroendocrine/immune connections are conserved across vertebrates and invertebrates (Ottaviani and Franceschi, 1996; Humphries and Yoshino, 2003; Cohen and Kinney, 2007; Adamo, 2008a,b; Ottaviani et al., 2008). The neuroendocrine and immune response show a combinatorial strategy where the repetitive use of a set of signaling molecules is shared by the immune and neuroendocrine systems for different purposes and functions. Interestingly, it is now believed that this cooperation has been acquired through evolution as a consequence of some extreme parsimonious strategies (Demas et al., 2010). It is well-known that invertebrates possess endocrine/neuroendocrine systems, as well as, an immune system, which in turn, releases stress hormones/neurohormones in response to stress stimuli, and when it has been discovered the presence of hormone receptors in immune cells associated to intracellular signaling pathways, it has been suggested that the effects of the stress response on immune system is adaptive (Adamo, 2008a).

In mollusks, insects, and crustaceans, stress hormones are responsible for modulating immune function via maintaining immunity function under a changing internal environment (Demas et al., 2010). This continuous cross talking at molecular, cellular, and physiological levels contributes to the enhancement of some immune functions, while suppressing others. The knowledge of this balancing is critical for understanding the adaptive function of stress hormones on immune function (Adamo, 2010, 2012). On the other hand there is a shortage of data available in literatures regarding the evolutionary origin of the synergic communication between the neuroendocrine and immune systems, except of limited reports on invertebrates (Ottaviani and Franceschi, 1996; Adamo, 2008a,b).

Cephalopods (squids, cuttlefish, octopods, and nautiluses) belong to a class of the Mollusca phylum and are exclusively marine organisms that occupies a variety of different habitats, thanks to a variety of adaptations. It is estimated that out of 1000 cephalopods species, 700 have been recorded living in all oceans of the world with the exception of the Black Sea, as well as spreading from shallow waters down to deep sea, inhabiting a wide range of ecological niches. In general, and due to cephalopods wide distribution and their ability to adapt quicker to environmental changes compare to many marine species, cephalopods play an important role in community organization and ecosystem function. According to Doubleday et al. (2016), over the past six decades, cephalopod populations showed an increasing trend in proliferation in response to changes in world's oceans. Cephalopods are prey and predators and any minor changes in their health or population composition could have unknown consequences on other populations, including those used for human consumption. Moreover, many cephalopod species are considered of economic importance for fisheries and excellent candidates for aquaculture (Jereb and Roper, 2005).

*Octopus vulgaris* is regarded as the “candidate” species in European aquaculture, mainly for its easy acclimatization to farming conditions, rapid growth and its good market value (Vaz-Pires et al., 2004). Recently the Directive 2010/63/EU requires appropriate care and maintenance of cephalopods for research purposes urging to develop proper knowledge and practice in terms of assessment of health and disease prevention of these animals. From now on, we will outline in this short review, the wealth of information available in cephalopods (mainly in *O. vulgaris*) to describe the role played by neuroendocrine and immune systems in response to stress-stimuli, and try to understanding the fine threads orchestrating the response to stimuli between endocrine, nervous, and immune systems. Developing an integrated approach that combines several fields such as neuroscience, physiology, immunology, ethology, ecology, and evolution will highlight the adaptive role of the different interactions and their possible role to shape behavior and host defense.

## Neuroendocrine response in cephalopod *O. vulgaris*

It is a well-known fact, that the endocrine system coordinates physiological processes via secretion of chemical messengers through certain organs, and that the nervous system, the other internal correlation system, plays a key role in coordinating these processes. These internal correlation systems allow the organism to respond to numerous exogenous and endogenous stimuli (environmental and physiological).

How these two systems cooperate regulating the neuroendocrine processes?

In the classical concept of input–output relations, stimuli coming from environment, as temperature, photoperiod, food, and partner's availability, are perceived by sensory organs and specific nervous areas receive, and decode the signals, and by doing so, generating internal responses that affect endocrine glands to release chemical messengers, which in turn bind to target organs. There is no doubt that the two systems work in different way as in messengers release mechanisms and the distance between release sites and target tissues. Interestingly, the messengers mode of action is restricted to one of the following types: fast neurotransmitters, slow neurotransmitters, neuromodulators, hormones, and paracrine factors (Webster et al., 2002).

In molluscs, the taxon in which endocrine gland first appeared, the nervous system is quite complex, exhibiting a different organization among classes with neurosecretory and nerve cells in the neurohemal areas and in endocrine glands (Ketata et al., 2008). This increasing complexity of neuroendocrine system has been classified as first, second, and third order control systems depending on the number of endocrine glands and target tissues (LaFont, 2000). The number of the neurosecretory organs and messenger molecules involved defines different level of complexity. In bivalves and gastropods, neurohormones secreted by nervous ganglia and gonads stimulates sexual maturation showing first and second order control systems, respectively (Smit et al., 1988; Nagle et al., 1989; Smith et al., 1996; Pazos and Mathieu, 1999; De Lisa et al., 2013), while cephalopods show a third order neuroendocrine regulatory system comparable to vertebrates HPG axe (LaFont, 2000; Ketata et al., 2008; De Lisa et al., 2012; Di Cosmo and Polese, 2014).

In *O. vulgaris* the chemical messengers involved in the cross-talk between nervous and endocrine system in reproduction control, are classified into three main categories: neurotransmitters, neuromodulators, and hormones considering their chemical properties and mechanism of action (Di Cosmo and Polese, 2014). Several neuropeptides, neurotransmitters, and hormones has been detected in the nervous lobes and endocrine glands controlling reproduction in *O. vulgaris* giving rise to the model recently proposed (Di Cosmo and Polese, 2014; Polese et al., 2015). In this model the nervous lobes that control the olfaction, the olfactory lobes, and the olfactory organs, are at the center of a dense network of molecules that provides connection to the nervous system and the endocrine glands; then they regulate the switch on-off between energy storage and reproduction. The question to be asked here is, how this regulation occurs?

The olfactory information coming from the environment determines: feeding choices, selection of mates and habitats, competitive interactions, and energy transfer within and among ecosystems. These informations are detected by olfactory organs that are connected by olfactory nerve to olfactory lobes. The olfactory lobes are responsible for modulating the olfactory organs to be more sensitive to food odors, especially during the energy storage period (from paralarva to adult), which in turn inhibits the activity of optic gland responsible for gonad maturation switching off reproduction. On the other hand, and during the reproductive period, the olfactory lobes modulate the olfactory organs to be more sensitive to sex odors switching on reproduction. This model, which links the nervous and endocrine systems, shows how the two systems are provide a coordinate response to a variety of stimuli coming from environment to optimize the animal fitness.

## Immune response in cephalopod *O. vulgaris*

It's well-documented that wild and reared cephalopods are affected by a variety of pathogens, as in the form of bacteria, protozoa, and metazoan parasites (Castellanos-Martínez and Gestal, 2013). Yet, research aiming at understanding cephalopods-pathogens interaction, especially in octopus and squid, is very limited, especially when compared to understanding immune response in other commercially important marine species, making it difficult to make generalizations across species (Ford, 1992; Pascual and Guerra, 2001; Castellanos-Martínez and Gestal, 2013; Troncone et al., 2014). Similarly, knowledge concerning immune and neuroendocrine systems, and the coordination between the two systems in response to different diseases, is fully unclear. All these facts lead us to focus on studying the immune system of cephalopods, particularly *O. vulgaris*, and to investigate the relationship between the immune and neuroendocrine responses in order to analyze how they modulate reciprocally the physiological responses required to ensure organism's health.

Cephalopods are considered “advanced” invertebrates and the only class to possess, among mollusks, a close circulatory system with one systemic, and two accessory hearts. The branchial hearts are involved in the production of hemocyanin (Schipp, 1987; Wells and Smith, 1987; Beuerlein et al., 1998, 2002). Cephalopods have an innate immune system similarly to other mollusks; their immune defense is mediated by activation of cellular factors as antibacterial peptides, whereas the immune system works on the basis of “cellular factors” (Troncone et al., 2014; Castillo et al., 2015). The cephalopod hemocytes respond by phagocytosis, encapsulation, infiltration, or cytotoxic activities to infections and destroy or isolate pathogens. The white body is the hematopoietic organ of cephalopods; this pair organ has glandular appearance, and is located behind the eyes, around the optic tracts, covered by the cranium, and it is responsible of hemocytes production (Cowden, 1972). In *Octopus hubbsorum* as demonstrated by Pascual et al. (2006) the hemocytes repaired the injured tissue by hook catches, moving and aggregating at the injured site. More interestingly, it has been reported that the cephalopod hemocytes are able to produce oxygen and nitrogen radicals as defense response; NO produced by *O. vulgaris* hemocytes seems to decrease with the increase of infection by *Aggregata octopiana* (Rodríguez-Domínguez et al., 2006). Furthermore, the cephalopod hemocytes can be used as an indicator of the organism's health (Ellis et al., 2011), since variations in their number and morphology has been related to parasitic infections (da Silva et al., 2008).

In addition to the role played by hemocytes, molecules dissolved in the serum (opsonins, agglutinins, lysozyme) also contribute to the immune response (Ford, 1992; Castellanos-Martínez and Gestal, 2013; Troncone et al., 2014; Castillo et al., 2015; Gestal and Castellanos-Martínez, 2015). In *O. vulgaris* and in *Octopus maya*, cell-free hemolymph humoral factors as lectins, are able to agglutinate oligosaccharides from pathogens (Rögener et al., 1987a,b; Alpuche et al., 2010).

Recently our group identified in *O. vulgaris* hemocytes crude methanolic acid extracts, a novel antibacterial activity, the first antimicrobial activity against the most clinically isolated human pathogens. A very low concentration of this extract was able to completely inhibit bacterial growth (Troncone et al., 2014). However, we characterized the hemocyte populations based on their morphological, cytofluorometric and functional properties developing a primary hemocytes culture on a novel culture medium. In this study we identified three hemocytes population by cytofluorometric analysis, R1, R2, R3 corresponding to a gradient of a differentiation stages supporting the theory that one blast type, hemoblast-like gives origin to hyalinocytes that further mature in granulocytes. Interestingly the granulocytes, which are fully mature hemocytes, corresponding to R3 population, represent the only cell type able to proliferate. However, granulocytes are very avid phagocytes, in respect to hyalinocytes contributing actively to defense response unlike hemoblast-like cells that lack the phagocitotic activity and the intracellular enzyme system associated with immunity response (Troncone et al., 2014).

The recent advances of omics era, has had its effects on the type of data generating from *O. vulgaris*; it provided interesting molecular bases for *O. vulgaris* immune system through transcriptomic, genomic and proteomic approaches (Castellanos-Martínez et al., 2014a,b; Albertin et al., 2015; Gestal and Castellanos-Martínez, 2015; and ongoing project of Moroz et al.^1^). A wide and different range of genes and molecules has been identified that is believed to be involved in the cephalopod immune defense with similarities to vertebrates' immune response.

Recent transcriptome analysis in *O. vulgaris* hemocytes indicated that about 3% of the predicted proteins, were actually immune function-related, as well as putative members of the complement, the Toll-like receptors (TLRSs) that were found to be highly conserved across evolution and responsible for a variety of responses to bacterial, viral, fungal infections, self-derived products, and bactericidal permeability-increasing protein (Rauta et al., 2013). Moreover, the peroxisome proliferator-activated receptors (PPARS), an anti-inflammatory factor able to interact with transcription factors involved in inflammation also has been identified (Gestal and Castellanos-Martínez, 2015). The recent findings of the specific transcripts sharing high similarity to Jaw1, a specific protein present in the lynphocytes B and T strongly suggest that similar molecules and mechanisms are conserved in phylogenetically distant animals.

Interestingly, differential analysis using high-throughput sequencing, to study coccidiosis infected *O. vulgaris* individuals, caused by parasite *A. octopiana*, resulted in identification of 312 genes that were differentially expressed (Gestal et al., 2007). Functional studies showed that the expression of TLR was up-regulated in octopus with high parasitic infection; on the contrary, peptidoglycan recognition patterns (PGRP), C1q binding protein and oxidative activity expressions, were downregulated (Castellanos-Martinez and Gestal, 2011). Octopus with high coccidian load shown an upregulation in cytoskeletal proteins as actin, filamin, and fascin due to an increases of phagocitotic activity (Castellanos-Martínez et al., 2014b), in particular fascin, known to directly contributing to the regulation of immune response (Feng and Walsh, 2004). All these findings, at the molecular levels, could be further used in identifying molecular markers for octopus's resistance to coccidiosis; and when all this is combined with transcriptomic and proteomic results, a better understanding of the immune system and its response to pathogens could be achieved.

**Figure 1:**
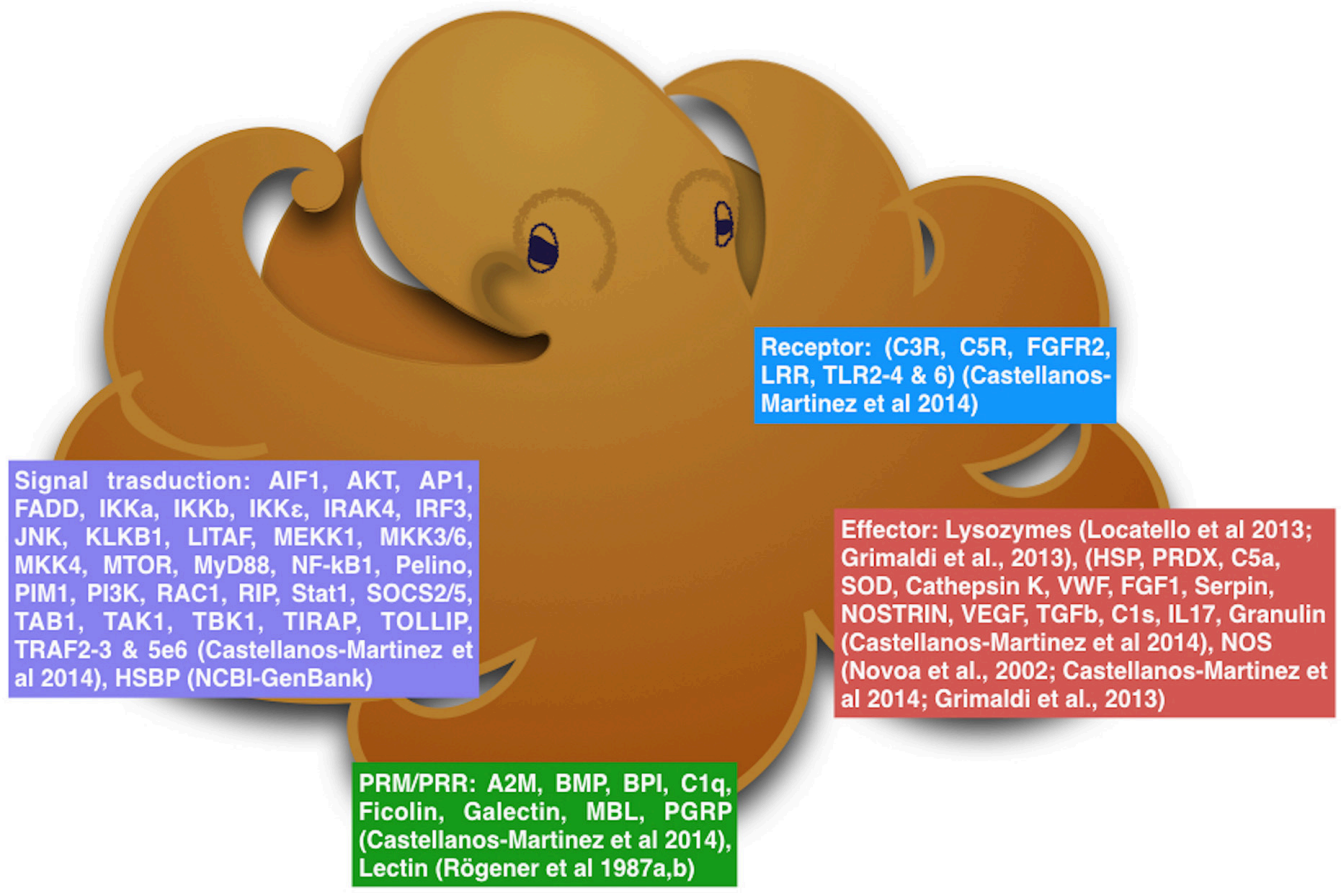
**The diagram reassumes the molecular mechanisms that octopus can use to protects itself from environmental stressors**.

First results coming from epigenetic approach revealed that the genome of *O. vulgaris* is widely methylated with no differences between the sex but with some differences in the mantle of specimens at different stage of maturity, probably due to the complex hormonal machinery that regulates the reproduction (Díaz-Freije et al., 2014; Di Cosmo and Polese, 2014; Polese et al., 2015). Interestingly but not surprising, the methylation pattern found in octopus paralarvae were higher than those observed in the adult animals (Díaz-Freije et al., 2014). *O. vulgaris* has a short life span, and octopus at the paralarval stage are considered very susceptible to environmental conditions as in, food availability and presence of predators. These environmental conditions might affect the gene transcription through epigenetic mechanisms influencing development of the immune system to face and improve survival and health. All these facts reveal that immune response in cephalopods could be more complex and evolved than expected (Figure 1; Castellanos-Martínez et al., 2014a).

## How neuroendocrine–immune systems cooperate in response to stress stimuli in *O.vulgaris*?

Throughout evolution, organisms always had a need to detect and respond to environmental conditions and challenges. Among animals, diversification in sensory and signaling systems has resulted in beneficial adaptations as well as variable sensitivity to different stresses. While there is a substantial evidence about the role played by the neuroendocrine crosstalk to regulate the fundamental physiological balancing between energy storage and reproduction in *O. vulgaris* (Di Cosmo and Polese, 2014; Polese et al., 2015), as well as many information regarding the immune system and defense (Troncone et al., 2014; Gestal et al., 2015), it is important to indicate that to our knowledge, and despite the fact that cephalopods possess a number of specializations as in, signaling molecules, appropriate receptors placed on specific cells, and mechanisms to transmit peripheral immune signals across the blood brain barrier, there are just a few data describing the interaction between the neuroendocrine and immune systems to respond to environmental challenges (Malham et al., 2002; Larson and Anderson, 2010).

**Figure 2:**
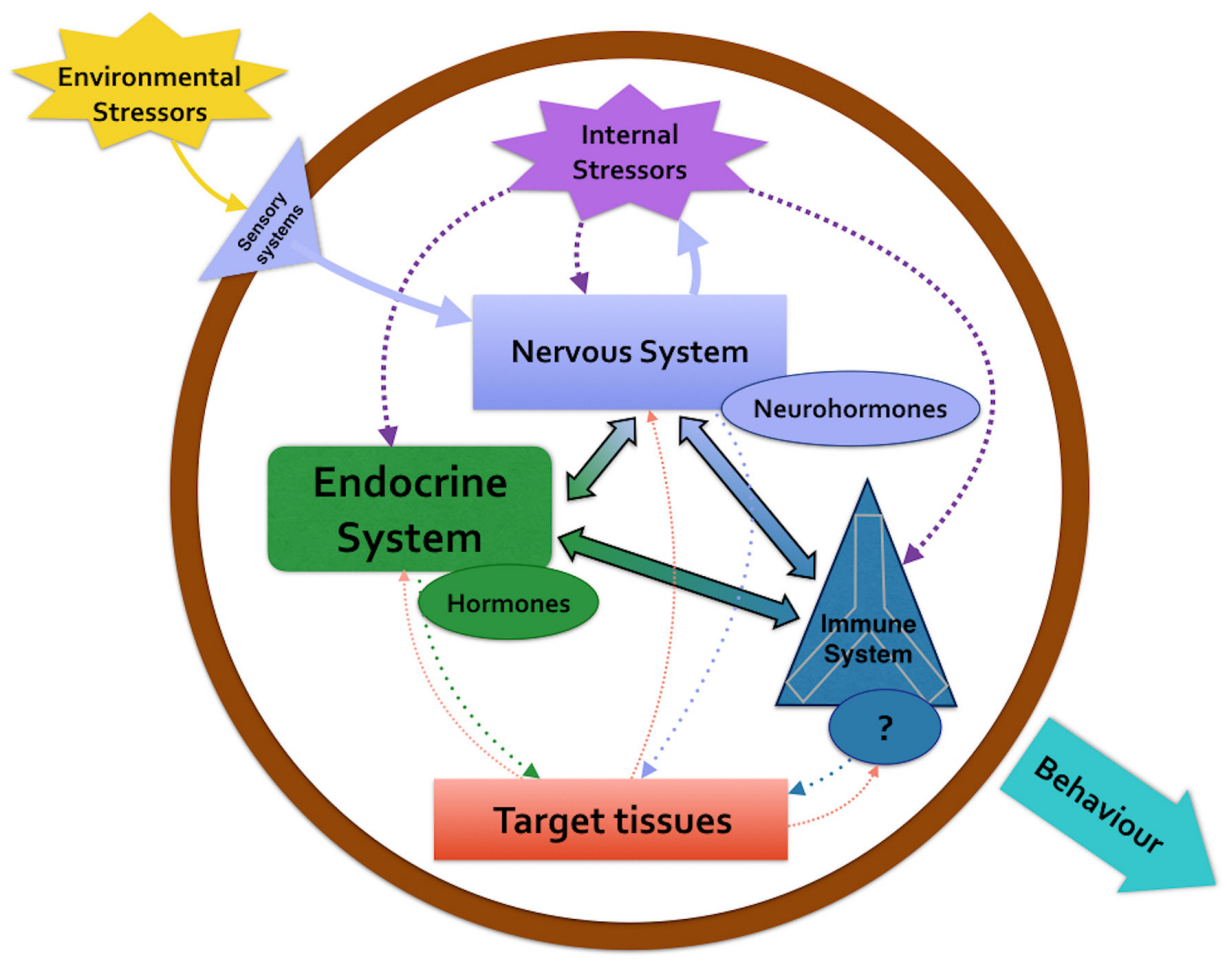
**The diagram describes the crosstalk among nervous system, endocrine system and immune system in response to an environmental stressor in *Octopus vulgaris***.

However, the work made by Larson and Anderson (2010) on fecal corticosterone level in captive *Enteroctopus dofleini* represents a useful tool to evaluate the connection between the neuroendocrine and immune system. They demonstrate by immunoassay that fecal corticosterone levels increased after administrating three stresses, including the adrenal gland stimulator ACTH. In vertebrates this hormone, as well as glucocorticoids, are secreted by pituitary or adrenal gland in response to stressful events and are considered stress hormone. In the last 20 years, the presence and the role played by the axe nervous system/subpedunculate area/optic gland/gonad resembling the vertebrate HPG axe has been widely demonstrated in *O. vulgaris*. The presence and function exerted by sex-steroid, neuro-steroids and their receptors shaping the adaptive responses strongly supports the release of corticosterone (Larson and Anderson, 2010; De Lisa et al., 2012; Polese et al., 2015). Octopuses exposed to different stressful stimuli, that negatively affect growth, reproduction, metabolism, osmoregulation, and immune ability, usually release corticoid stress hormones; the corticosterone that could be released through the nervous system/sub-pedunculate area/optic gland represents a link between neuroendocrine and immune systems in response to stresses (Figure 2; Di Cosmo and Di Cristo, 1998; Di Cosmo et al., 2001, 2002; De Lisa et al., 2012; Di Cosmo and Polese, 2013; Polese et al., 2015).

In general, and due to the complexity of animal responses to environmental and chemical factors needs more attentions form workers in this field, to evaluate how the neuroendocrine and immune systems interact each other to better understand the animal heath at individual and population levels.

## Author contributions

AD and GP equally contributed to this mini review.

## Funding

This work was supported by grants from the Italian Ministry of University and Research: FFO2014, FFO2015.

### Conflict of interest statement

The authors declare that the research was conducted in the absence of any commercial or financial relationships that could be construed as a potential conflict of interest.

## Footnotes

^1^http://neurobase.rc.ufl.edu/cephalopods.
